# Smartphone app-based interventions on physical activity behaviors and psychological correlates in healthy young adults: A systematic review

**DOI:** 10.1371/journal.pone.0301088

**Published:** 2024-04-05

**Authors:** Zihao He, Mohamed A. Hassan, Pablo Saiz-González, Suryeon Ryu, Ronghui Wang, Zan Gao

**Affiliations:** 1 School of Sport Science, Beijing Sport University, Haidian, Beijing, China; 2 School of Kinesiology, University of Minnesota-Twin Cities, Minneapolis, Minnesota, United States of America; 3 Department of Methods and Curriculum, Physical Education College for Men, Helwan University, Cairo, Egypt; 4 Faculty of Teacher Training and Education, University of Oviedo, Asturias, Spain; 5 Department of Kinesiology, Recreation, and Sport Studies, The University of Tennessee, Knoxville, Tennessee, United States of America; Universiti Malaya, MALAYSIA

## Abstract

**Background:**

The issue of low physical activity (PA) levels among the youth is a longstanding concern. Smartphone applications offer a promising avenue for delivering interventions that are both accessible and engaging. Up to now, there appears to be a gap in the literature, with no systematic reviews assessing the efficacy of smartphone apps in encouraging increased physical activity among healthy young adults.

**Objective:**

To synthesize the effects of a smartphone app-based intervention on PA and PA-related psychological correlates in healthy young adults (18–35 years old).

**Methods:**

A search was conducted on eighteen databases: PubMed, Medline, Web of Science, SPORTDiscus, Scopus, Academic Search Premier, Communication and Mass Media Complete, Article First, Biomed Central, BioOne, EBSCOHost, JSTOR, ProQuest, SAGE Reference Online, ScienceDirect, SpringerLink, Taylor&Francis, and Wiley Online. The search covered the period up until December 2023. This research included all randomized controlled trials (RCTs) that evaluated the effectiveness of smartphone app-based interventions on PA and PA related psychological outcomes in healthy young adults. The overall impact was determined by vote counting based on the direction of effect and aggregating p values. The quality of the evidence was evaluated using an 8-item scale. This study has been registered in the PROSPERO database with the identification number CRD42023390033.

**Results:**

A total of 8403 articles were retrieved, and based on the predefined inclusion and exclusion criteria, seven articles were selected for inclusion. Among these articles, four high-quality RCTs were identified, and the results of vote counting and combining p values methods suggested that smartphone-based app interventions did not demonstrate significant effectiveness in improving PA and PA-related psychological outcomes. However, some improvements were observed. The analysis results, which were categorized into fitness apps and health apps based on the characteristics of the interventions, also failed to demonstrate significant intervention effects.

**Conclusion:**

The findings indicate that, currently, there are no significant effects of smartphone app interventions on improving PA and PA-related psychological outcomes in healthy young adults aged 18–35 years. It is important to note that these findings should be interpreted with caution due to the limited number of included studies. Future research should focus on employing high-quality study designs to determine the true effects of interventions and analyze various smartphone app interventions. These analyses should encompass different app characteristics (e.g., fitness app and health app), various combinations (e.g., fitness app alone and fitness app in combination with other interventions), diverse intervention goals (e.g., PA and PA along with other outcomes), and multiple intervention characteristics (e.g., frequency and duration).

## 1. Introduction

Insufficient engagement in physical activity (PA) presents a notable risk factor for a range of health complications, encompassing cardiovascular disease, cerebrovascular disorders, metabolic conditions, cancer, mental health disorders, and premature mortality [1–4]. According to a study from *The Lancet Global Health*, if the rate of physical inactivity (PI) remains the same worldwide, there would be approximately 500 million additional cases of preventable non-communicable diseases between 2020 and 2030, resulting in associated medical cost of $520 billion [5]. The *Global status report on physical activity 2022*, released by the World Health Organization (WHO), presents that an estimated 1.4 billion adults worldwide, accounting for around 27.5% of the global adult population, do not meet the guidelines for engaging in at least 150–300 minutes of moderate PA or 75–150 minutes of vigorous PA per week. This number has remained largely unchanged over the years [6].

The period of young adulthood, particularly while attending college, represents a critical phase in the transition from adolescence to adulthood [7]. This period is characterized by a decline in PA among the majority of students and a higher likelihood of developing unhealthy lifestyle habits, influenced by factors like personal circumstances and psychological adjustments [8, 9]. Consequently, this can give rise to various health concerns, including reduced physical fitness, obesity, and depression.

Despite the efforts of numerous studies in the field, effective strategies to improve PA among healthy young individuals remain elusive. Most existing intervention options are costly, difficult to replicate, and have limited sustainability [10–12]. Therefore, there is a need for innovative PA promotion strategies that cater to the interests of young adults. The *Global Status Report on Physical Activity 2022* emphasizes the transformative impact of digital and mobile technologies on healthcare delivery, presenting new opportunities for promoting PA [6]. Mobile technology has made significant advancements in the health sector over the past decade. One potential strategy to leverage these advancements is the utilization of smartphone apps as intervention tools. Statistics project that by 2023, approximately 6.92 billion people worldwide, accounting for around 86.29% of the global population, will be smartphone users [13]. Moreover, as new technologies continue to progress, mobile apps are evolving to serve multiple functions, addressing various aspects of daily life. These apps increasingly prioritize enhancing user experience and incorporate intelligent features like artificial intelligence [14] and virtual reality [15]. For example, smartphone apps offer several advantages, including affordability, widespread usage, intelligence, and interactive capabilities, which make them promising tools for intervention aimed at improving PA among adults.

Several prior systematic reviews have examined the impact of app-based interventions on improving PA. For example, Rodríguez-González et al. conducted a systematic review of systematic reviews to summarize the scientific evidence and found that two systematic reviews reported a significant improvement in PA, while seven reported positive effects [16]. This suggests the potential effectiveness of apps in enhancing individuals’ PA. When specifically analyzing the adult population, four systematic reviews reported significant improvements in PA with app interventions: Kim et al. [17] (standardized mean difference (SMD) = 2.59, 95% CI 1.00 to 4.18), Feter et al. [18] (steps per day [SMD = 0.18, 95% CI 0.01 to 0.35] and minutes per day [SMD 0.31, 95% CI 0.01 to 0.60]), Silva et al. [19] (steps per day [weighted mean difference = 1579.04, 95% CI 454.04 to 2703.38]), and Pradal-Cano et al. [20] (13 out of 14 studies reported the effectiveness of apps in increasing PA). However, two reviews did not show a significant effect: Romeo et al. [21] (mean difference (MD) = 476.75, 95% CI -229.57 to 1183.07) and Yerrakalva et al. [22] (506 steps/day, pooled MD 95% CI -80 to 1092). These inconsistent findings can be attributed to variations in the characteristics of the populations studied across the systematic reviews. Some reviews included adults (≥18 years) [18, 20, 21], young adults (18–35 years) [17], and older adults (≥55 years) [22]. Additionally, it is important to note that all adults included in these reviews had specific medical conditions such as obesity, cardiovascular disease, type 2 diabetes, stroke, and breast cancer. Hence, the efficacy of app interventions in different medical conditions, particularly among healthy young adults, who are often overlooked, remains uncertain.

Young individuals who are in good health and actively strive to increase their PA levels can potentially prevent health issues and reduce their future socioeconomic and medical burden. Encouragingly, there have been experimental studies investigating the impact of app-based interventions on improving PA in healthy young adults [23–29], and new studies have emerged in recent years [23, 26, 27]. While some studies have found no significant effect of the app intervention [24, 26, 28], others have reported significant improvements in PA among adults [23, 25]. These findings suggest that the effectiveness of current interventions in promoting PA among healthy young adults is somewhat inconclusive. Therefore, the objective of this review is to comprehensively synthesize published studies to summarize the effects of smartphone app interventions on enhancing PA in healthy young adults.

## 2. Methods

This review was conducted in accordance with Preferred Reporting Items for Systematic Reviews and Meta-Analyses (PRISMA) Guidelines [30]. A completed checklist can be found in S1 Table. The study protocol was registered in PROSPERO (registration number: CRD42023390033).

### 2.1. Information Sources

Literature searches were conducted with eighteen databases, including PubMed, Medline, Web of Science, SPORTDiscus, Scopus, Academic Search Premier, Communication and Mass Media Complete, Article First, Biomed Central, BioOne, EBSCOHost, JSTOR, ProQuest, SAGE Reference Online, ScienceDirect, SpringerLink, Taylor&Francis, and Wiley Online, as of December 2023. We also carried out a ‘snowball’ search to identify additional studies by searching the reference lists of publications eligible for full-text review to identify and screen studies citing them.

### 2.2. Search strategies

Two known relevant studies were used to identify records within databases [23, 27]. Candidate search terms are identified by looking at the words in the title, abstract, and subject indexing of these records. A draft search strategy was developed using these terms, and other search terms were identified from the results of the strategy. We also identified search terms through subject heading terms (MeSH) in Medline. The search strategy was validated by testing whether it could identify two known related studies. The development of the search strategy was determined by three researchers (P.S.-G., M.H., Z.H.) and the searches were carried out separately. In case of disagreement, a fourth researcher (Z.G.) coordinated to resolve the problem. The search term included adult and (“app” OR “mobile phone” OR “smartphone” OR “smart phone” OR “cellphone” OR “cell phone” OR “mHealth” OR “mobile health”) and (“physical activity” OR exercise OR step OR inactivit OR “sedentary behavior” OR “screen time” OR “screentime” OR “sitting time”) and (RCT or CCT). The search strategy was adapted to the search language of each database (S2 Table).

### 2.3. Study selection criteria

Inclusion and exclusion criteria were developed according to PICOS (population, intervention, comparison, outcomes, and study) principles [31].

(1) Population: participants were healthy young adults (mean age of 18–35 years), excluding non-healthy adults (e.g., diabetes, hypertension, overweight, obesity, etc.). (2) Intervention: primary intervention was based on smartphone apps, excluding interventions from other smartphone features such as short message service (SMS). Non smartphone interventions such as wearable devices, computers, and tablets are also excluded. (3) Comparison: comparison was no-intervention routine care, waiting list, or intervention did not include mobile technology such as an app or wearable device. (4) Outcomes: we extensively included experimental studies reporting PA related outcomes, including PA, PA behaviors, psychological outcomes of PA, etc; Research that only reported physical fitness was not included. Full text was written in English. If there were no PA related results reported, we contacted the corresponding author via email and requested more information, including key data. (5) Study design: RCT, controlled clinical trial was included.

### 2.4. Data collection process

The retrieved literature was imported into the online literature management tool *Rayyan* [32] and duplicate studies were removed. The titles and abstracts were then screened during the primary screening process to remove obviously irrelevant literature (Z.H.). Three authors (P.S.-G., M.H., Z.H.) individually screened the remaining literature, and those identified as eligible were read in full.

In addition, one reviewer (Z.H.) manually reviewed the reference lists of relevant original studies and review studies to add any literature that may have been missed during the initial search. If disagreements arose during the search process, the reviewers discussed them, and if the dispute remained unresolved, the other reviewer (Z.G.) was contacted for resolution.

We designed a data extraction form that was utilized by two researchers (M.H. and Z.H.) to extract information from the included studies. The information was then entered into Microsoft Word tables. The extracted information included author’s name (publishing date), country of study, study purpose, sample and design, and intervention (type, duration, frequency). In addition, any measure of PA behaviors and psychological correlates were eligible for inclusion. The results were reported as cumulative time of PA, metabolic equivalent, steps, etc. We expect individual studies to report data on multiple PA results. Specifically, a study may report the following results: (1) for multiple structures related to PA, such as total PA (TPA) and specific intensity of PA (e.g., moderate to vigorous intensity PA (MVPA), light intensity PA(LPA), etc.); (2) Measure at multiple time points, such as 1 month, 3 months, and 6 months. If multiple PA outcomes were reported, we selected all outcomes with same variable type to be included in the analysis and report (e.g., TPA, TPA, LPA, etc.) and use standardized mean differences (SMD) for processing [33]. When there were several publications from the same project, the study with the longest follow-up was selected; if there was no intervention during the follow-up, the result of the last intervention was selected as statistical analysis data. In case of any inconsistencies, the author (Z.G.) was consulted for resolution.

### 2.5. Risk of bias in individual studies

The risk of bias for each study was evaluated using an 8-item scale. Depending on prior research [34–36], each item was rated as positive (+), negative (−), or not applicable (NA). Positive (+) scores were assigned when the study clearly met the category, while negative scores (−) were given when the category was missing or inaccurately reported. The sum of all positive (+) scores was used to determine the final score. Studies with a final score of five or higher (median score) were considered to have a high-quality design and a low risk of bias, whereas studies with a score less than five were considered to have low quality and high risk of bias. Two researchers (M.H. and Z.H.) were involved in the assessment process independently. Then, discrepancies were consulted by a third author (Z.G.).

### 2.6. Statistical analysis

STATA (produced by StataCorp, College Station, TX, USA. https://www.stata.com/) was used for statistical analysis. Given the substantial heterogeneity observed among the included studies in terms of study design, intervention characteristics, and outcomes, meta-analysis was not conducted in this study. Instead, other statistical synthesis was adopted [30]. According to suggestion of Cochrane Handbook for Systematic Reviews of Interventions (Version 6.4): Chapter 12 (Synthesizing and presenting findings using other methods), summarizing effect estimates, vote counting based on direction of effect, or combining P values are acceptable methods [37]. Due to the variety types of outcomes and different statistical tests used across the studies, this study used vote counting based on direction of effect and combining p values [37]. The utilization of two distinct methods was driven by the recognition that certain datasets might align more closely with one approach over the other, thus employing both strategies allows for a more nuanced interpretation of the findings. The research was organized initially by the unique features of the interventions they examined, distinguishing between fitness and health applications, to assess both the collective outcomes and the results specific to each intervention category.

Vote counting based on direction of effect refers to the evaluation system approach employed by Khoo et al. [38] and Rodríguez-González et al. [36] to analyze the effectiveness of smartphone apps in enhancing PA in healthy young adults. This approach was used to assess the effectiveness of the interventions across various categories. To synthesize conclusions about effectiveness based on the best available evidence, a rating system of levels of evidence was utilized. The level of evidence included strong, moderate, limited, inconclusive, and no effect, which were determined based on study design, methodological quality, and sample size. Conclusions were drawn based on the consistency of findings at the highest level of quality achievable. If at least two thirds (66.6%) of the studies reported significant results in the same direction, the overall results were considered consistent [38–40].

Combining p values to present the results through albatross plot. Albatross plot allows for approximate examination of potential intervention effects with minimal reported results in the study [37]. This graph only requires the double-sided p-value, sample size, and effect direction (or equivalent, single-sided p-value and sample size) for each result. This graph is a scatter plot of the study sample size and double-sided p-values, where the results are separated by the direction of the effect. Overlaid on the graph is the “effect size outline”. These profiles are specific to the data types (e.g., continuous, binary) and statistical methods used to calculate p-values. Initially, all methods require the computation of standardized metrics, followed by the application of comprehensive analytical techniques. This approach ensures the data are formatted appropriately for either presentation or integration into broader meta-analyses [37]. The formulas for the mean and standard deviation (SD) pre- to post-change values were as follows: ‘Mean_change_ = Mean_post_−Mean_pre_’ and ‘SD_change_ = SQRT [(SD_pre_2 + SD_post_2)—(2×Corr×SD_pre_×SD_post_)]’, according to the Cochrane Collaboration Handbook guidelines, where the Correlation Coefficient was set to 0.5. SMD were calculated in this study due to the use of several measuring tools. Combining p-values will adopt Fisher’s proposed method, which combines the P values from statistical tests across k studies using the formula: $X^{2}=-2\sum_{i=1}^{k}ln\left(P_{i}\right)$. Using unilateral p-values because they contain information about the direction of the effect. Bilateral p-values without directional information must first be converted to unilateral P-values. If the effect is consistent with the direction assumption, the unilateral P-value is calculated as: $P_{1-sided}=\frac{P_{2-sided}}{2}$, otherwise, $P_{1-\;sided\;}=1-\frac{P_{2-sided}}{2}$. Albatross plots were created to provide a graphical overview of the data for interventions with more than five or more data points for an outcome [37].

As this study is not a meta-analysis, graphical and statistical approaches cannot be used to assess reporting biases (e.g. funnel plot, Egger test). According to the Cochrane Handbook [41], authors should not ignore any missing results when drawing conclusions in this situation in the context of qualitative research. Therefore, we will use Grading of Recommendations Assessment, Development and Evaluation (GRADE) to evaluate potential reporting biases and discuss them [42]. There are two items relating to risk of reporting biases, including study limitations (including selective outcome reporting) and publication bias. Study limitations evaluation level is divided into no serious limitations, do not downgrade; serious limitations, rate down one level (i.e., from high to moderate quality); very serious limitations, rate down two levels (i.e., from high to low quality or moderate to very low). Publication bias evaluation level is divided into undetected and strongly suspected [42]. Please refer to S3 Table for evaluation criteria.

## 3. Results

**Fig 1:**
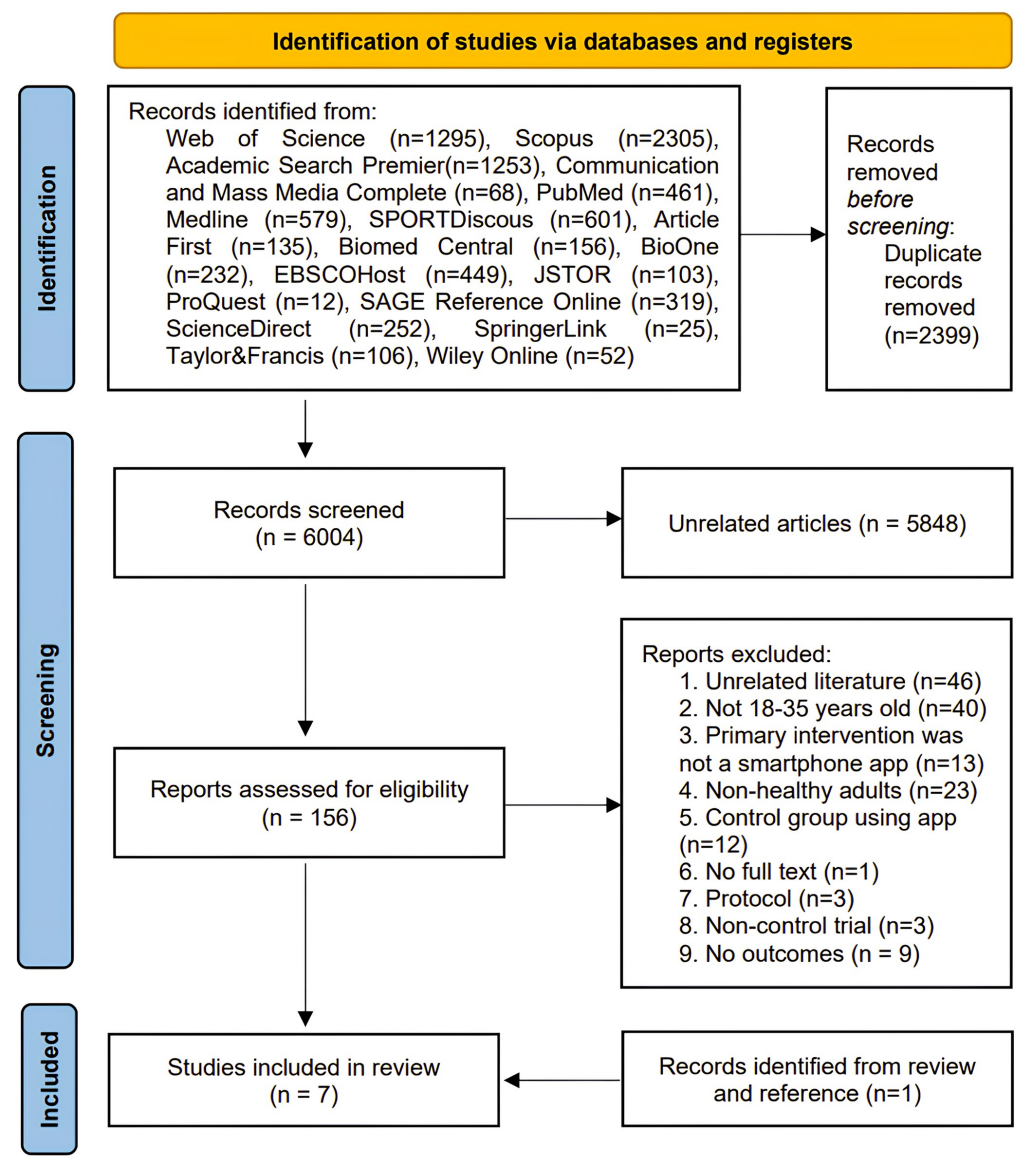


A comprehensive search of the eighteen databases yielded a total of 8403 relevant literature. Following the removal of 2399 duplicate articles, 6004 articles remained. These articles then underwent a preliminary screening, resulting in the exclusion of 5848 unrelated articles. The remaining 156 articles were subjected to full-text reading, which led to the exclusion of 150 articles. An additional article meeting the inclusion criteria was obtained by reviewing the references [24]. Ultimately, a total of 7 articles were included in the analysis [23–29]. The process of screening the literature is presented in Fig 1.

### 3.1. Study characteristics

The included literature was published between 2014 and 2023, with six articles published after 2018. The distribution of countries included USA (n = 2) [23, 27], UK (n = 1) [24], China (n = 1) [29], Belgium (n = 1) [28], Italy (n = 1) [25], and Indonesia (n = 1) [26]. The total sample size was 1622, with 835 participants in the intervention group and 787 participants in the control group. The mean age of the participants ranged from 18 to 25 years (Table 1). In terms of intervention characteristics, four studies used fitness apps [23, 25, 27, 28], which targeted mainly exercise and fitness. Three studies utilized health apps [24, 26, 29], which aim to enhance not only exercise and fitness but also other lifestyle aspects such as diet and nutrition. The intervention duration ranged from 2 weeks to 24 weeks. The outcomes reported in the literature included PA, PA behaviors, PA-related psychological outcomes, with secondary outcomes such as retention rate and satisfaction (Table 1).

**Table 1:** Synthesis of studies to generate a literature review.

Authors (year)	Study Purpose	Sample and design	Intervention	Control	Measurement	Findings
Epton (2014) [24]	To assess the efficacy and cost-effectiveness of a theory-based online health behavior intervention for new university students.	Total n = 1445 (844 female); intervention group n = 736 (453 female); control group n = 709 (391 female); M_age_ = 18.9; RCT; 24 weeks.	Participants were asked to log onto app and view online resources that included theory-based information related to each of the four target health behaviors (i.e. text, videos and links to further information) as well as a planner containing instructions that formed the intention to implement.	Daily life.	Outcomes: TPA Tools: The Short Form of IPAQ	TPA at 6-month did not differ significantly between the two arms.
Simons (2018) [28]	To examine the effect and process evaluation of the newly developed evidence and theory-based smartphone app “Active Coach” on Active Lifestyle among lower educated working young adults.	Total n = 130 (67 female); intervention group n = 60 (25 female); control group n = 70 (42 female); M_age_ = 25±3; cluster RCT; 9 weeks.	Users of the app receive tailored information about their goal, tips, and facts through notifications on their smartphone and messages in the Active Coach app. To ensure all-day and automatic self-monitoring of PA, the app works in combination with a wearable activity tracker, the Fitbit Charge.	Control group received a printed brochure with generic information and tips about a physically active lifestyle.	Outcomes: TPA, MVPA, Steps, PA-related psychological correlates Tools: Actigraph GT3X+accelerometers, IPAQ, The Dutch IPAQ, psychosocial outcomes were assessed during the interview.	No significant intervention effects were found for objectively measured PA, self-reported PA, and self-reported psychosocial correlates (all *P*>.05); significant time effects showed a decrease in the objective LPA, TPA, and number of steps from baseline to posttest; in both groups, perceived benefits significantly decreased from baseline to posttest and to follow-up, while knowledge regarding the PA recommendations significantly increased from baseline to posttest and tofollow-up.
Gabbiad ini(2019) [25]	To test whether the use of fitness apps for daily steps tracking could positively influence people’s health behavior.	Total n = 78 (69 female); M_age_ = 19.94±1.36; cluster RCT; 2 weeks.	All the adopted apps count daily steps, burned calories, and active time, altering users with notifications about daily achievements directly on the smartphone.	No information regarding mobile apps was given for participants in the control group.	Outcomes: attitudes, PBC scores, self-reported behavior Tools: adapted version of the attitude regarding physical activities for health and fitness scale, adapting the original items proposed by perugini and bagozzi	Participants in the experimental condition reported more favorable attitudes and higher PBC in the post-test compared to the pre-test, but not in control condition; participants using fitness app reported to have walked more in the post-test compared to the pre-test, but not control condition.
Wang (2020) [29]	To evolve traditional health intervention by using integrated methods based on social media and multiple mobile tools.	Total n = 110 (19 female); intervention group n = 87 (9 female); control group n = 23 (10 female); M_age_ = 22 ± 2 and 21 ± 4; RCT; 21 days.	Staffs used the WeChat app and its plugin Zhishi mini-program for health education, diet and PA supervision during 21 days.	Control group downloaded the WeChat app onto their mobile phones without any health behavior reminders.	Outcomes: PA Tools: IPAQ	An enhanced PA level was observed in intervention group, 48 participants were at a low PA level in baseline, and 26 of them moved to a higher level after the 21 days intervention (*P* = 0.004). About 60.9% subjects were satisfied with the whole program and 64.4% would like to join the program again.
Pope (2020) [27]	To evaluated the feasibility of combining a smartphone application and theoretically-based, social media-delivered health education intervention to improve college students’ health behaviors and outcomes.	Total n = 44 (32 female); intervention group n = 22 (16 female); control group n = 22(16 female); M_age_ = 21.6; cluster RCT; 10 weeks	Using MapMyFitness smartphone application to log and track PA and receive twice-weekly SCT-based health education tips by Facebook.	Control group were only provided instructions regarding how to access a separate, but content-identical Facebook group, with a directive to not use any mHealth app during the study.	Outcomes: intervention interest,use/acceptability, retention, feasibility, MVPA Tools: questionnaire; a mix of opened-ended and Likert-type survey; Actigraph GT3X accelerometers; a 9-question self-efficacy measure; a 5-question Measure; a modified 9-question scale	Retention: > 95%; at 5 weeks, the experimental group had a slight 2.6%-increase MVPA/day (1 minute increase), but this increase was not sustained at 10 weeks. Comparison participants demonstrated a consistent decrease in MVPA/day over time, with a 12.6%-decrease observed from baseline to 10 weeks (5.6 minutes decrease). For social support, 21.0% and 15.4% increases in the experimental and comparison groups, respectively; Both groups had a 4.8%-decrease in perceived barriers to PA, with both groups improving to a point where they “Disagreed” with most of the listed circumstances acting as barriers to PA participation.
Al-Nawaiseh(2022) [23]	To improve step-counts and body weight using an m-Health mobile app among university students.	Total n = 114 (92 female); intervention (n = 56); control (n = 58); M_age_ = 21.12±2.2; RCT; 12 weeks baseline, and follow-up measures.	Received information about PA goals through SMS/emails. They used Pacer pedometer app to record their daily steps. Participants were encouraged to monitor their steps and feedback through app.	Received information related to daily recommended PA and benefits of walking regularly. No observation, interaction, or app usage.	Outcomes: Daily step-count Tools: pacer pedometer app	There was a significant difference in step count between baseline and follow-up measures in intervention group. There is significant difference in follow-up measures between intervention and control groups.
Kuston (2023) [26]	To investigate a pilot design focusing on developing technology-supported physical education course for increasing university students’ physical activity levels.	Total (n = 39); intervention group (n = 22); control group (n = 17); pilot study; 16 weeks.	The intervention contains lectures taught using a prototype including learning management system (PESSPA) and smartphone application (PESAPA).	The control groups received traditional PA course with no technological integrative.	Outcomes: TPA, MVPA, PA motivation, PA knowledge Tools: IPAQ; the behavioral regulation exercise questionnaire (BREQ-2); self-developed physical activity quizzes.	The technology-supported physical education course has positively influenced PA levels but did not reach a significant level. In addition, small-to-medium effect sizes between intervention and control groups in motivation outcomes and physical activity knowledge in favor of the intervention group.

Abbreviations: IPAQ: International Physical Activity Questionnaire; LPA: Light Intensity Physical Activity; MVPA: Moderate-to-Vigorous Intensity Physical Activity; PA: Physical Activity; PBC: Perceived Behavioral Control; PESAPA: Physical Education Supporting App for Physical Activity; PESSPA: Physical Education Supporting Site for Physical Activity; RCT: Randomized Control Trial; SCT: Social-Cognitive Theory; SMS: Short Messaging Service; TPA: Total Physical Activity

### 3.2. Quality and risk of bias assessment

Table 2 presents the quality and risk of bias assessment of the seven experimental studies included in this review. Four (57%) of the studies demonstrated high methodological quality, scoring of five or more on the overall assessment. It is important to note that group randomization was not feasible in one of the controlled trial [29]. Three studies received negative scores in both the categories of missing data and follow up analysis, contributing to their low risk of bias assessment scores [25, 26, 29]. Furthermore, only one study had an intervention duration of 6 months or longer [24].

**Table 2:** Quality and risk of bias assessment.

Articles	(1)	(2)	(3)	(4)	(5)	(6)	(7)	(8)	(9)	Score
Simons et al. [28]	+	+	+	+	+	+	+	+	-	8
Gabbiadini et al. [25]	+	+	+	+	-	-	-	-	-	4
Pope & Gao [27]	+	-	+	+	+	-	-	+	-	5
Al-Nawaiseh et al. [23]	+	+	+	+	+	-	+	-	-	6
Epton et al. [24]	+	+	+	+	-	+	+	+	+	8
Kuston et al. [26]	NA	NA	+	-	-	-	+	+	-	3
Wang et al. [29]	-	+	+	+	+	-	-	-	-	4

Note: (1) randomization was performed and adequately explained; (2) there was a control group and comparative analyses were performed between it and the intervention group; (3) pre-post analyses were performed for outcome variables; (4) dropouts did not exceed 30%; (5) statistical differences were reported at baseline and groups were comparable on outcome variables; (6) missing data were reported and considered for statistical analysis; (7) power analysis was performed; (8) validity and reliability of instruments were reported; (9) follow-up analysis was performed at 6 months or more. “+” refers to positive; “-” refers to negative; “NA” refers to not applicable. “+/-" represent significant improvements found in some measures while no significant effects were found in other measures.

### 3.3. Strength of evidence

In the included studies, raw data were extracted into coding tables, and the data were converted into statistical data required for vote counting and combining p values [37]. Please refer to S4 Table for the raw data.

#### 3.3.1 Vote counting based on direction of effect

This review encompasses four high-quality RCTs [23, 24, 27, 28]. Among these studies (Table 3**)**, two indicated that app-based interventions did not yield a significant improvement in PA, one reported a significant increase in daily step count, and one did not include a comparison between groups. Additionally, three high-quality studies conducted pre- and post-intervention comparisons within groups. Al-Nawaiseh et al.’s study revealed a significant enhancement in daily step count following the intervention [23], while Pope et al.’s study demonstrated an improvement in MVPA at week 5, although it was not sustained until week 10 [27]. Simons et al. observed decreases in LPA, TPA, and daily step count among the intervention group after 9 weeks, while MVPA increased, albeit not significantly [28]. In summary, the smartphone app-based interventions did not produce a significant effect on improving PA in healthy young adults, although they did exhibit some degree of improvement.

**Table 3:** PA direction of effect results sorted by risk of bias and type of apps.

Author(year)	Sample	Type of app	Quality(scores)	Outcome	Direction of effect
Simons (2018)	130	Fitness app	8	TPA	Favours control
				MVPA	Favours control
				Daily step	Favours control
Al-Nawaiseh (2022)	114	Fitness app	6	Daily step	Favours intervention*
Pope (2020)	44	Fitness app	5	MVPA	Favours control
Gabbiadini (2019)	78	Fitness app	4	Walking	Favours intervention*
Epton (2014)	1445	Health app	8	TPA	Favours intervention
Wang (2020)	110	Health app	4	Number of low PA	Favours control
Kuston (2023)	39	Health app	3	TPA	Favours intervention
				VPA	Favours control
				MPA	Favours intervention
				Walking	Favours control

*P < .05 considered statistically significant.

Abbreviations: LPA: Light Intensity Physical Activity; MVPA: Moderate-to-Vigorous Intensity Physical Activity; TPA: Total Physical Activity.

In relation to PA-related psychosocial variables (Table 4), one high-quality study [28] conducted a between-group comparison and did not find any significant changes in self-reported psychosocial variables, including benefits, barriers, self-efficacy, intention, knowledge, and social support. However, within the pre- and post-intervention group, PA knowledge showed a significant improvement, while PA benefits exhibited a significant decrease. Similar results were observed in several other studies. Pope et al.’s study demonstrated a 21% reduction in barriers after the intervention, but no significant changes in self-efficacy and social support [27]. Moreover, most studies reported high retention rates (> 70%), and participants expressed higher satisfaction and a preference for the app-based interventions. Overall, there was an improvement in PA-related psychosocial outcomes, particularly in PA knowledge, but the extent of improvement in other areas was not statistically significant.

**Table 4:** PA psychological correlates direction of effect results sorted by risk of bias and type of apps.

Author(year)	Sample	Type of app	Quality(scores)	Outcome	Direction of effect
Simons (2018)	130	Fitness app	8	Benefits	Favours control
				Barriers	Favours control
				Self-efficacy	Favours control
				Intention	Favours control
				Knowledge%(correct answer)	Favours control
				Social Support	Favours control
Pope (2020)	44	Fitness app	5	Self-Efficacy	Favours control
				Social Support	Favours intervention
				Enjoyment	Favours control
				perceived Barriers	Favours control
				Outcome Expectancy	Favours control
Gabbiadini (2019)	78	Fitness app	4	attitudes	Favours intervention*
				PBC scores	Favours intervention*
Kuston (2023)	39	Health app	3	Amotivation	Favours control
				knowledge	Favours intervention

*P < .05 considered statistically significant.

Abbreviations: PBC: Perceived Behavioral Control.

*3*.*3*.*1*.*1 Fitness app*. There are three high-quality RCTs (Table 3). In the study by Al-Nawaiseh et al., participants received information about their PA goals via SMS/email, and their daily step counts were recorded using the app named Pacer. They were encouraged to monitor their steps and provide feedback through the app. Significant improvements in step counts were found in the intervention group compared to the control group [23]. However, no significant improvements were seen in the study by Simons et al., where participants received tailored information about their goals, tips, and facts via notifications in the Active Coach app, combined with the use of Fitbit Charge [28]. The results showed no significant changes in LPA, MVPA, TPA, or step counts in the intervention group compared to the control group. Furthermore, even after intervention, TPA, LPA, and step count significantly decreased in the intervention group. In other studies focusing on within-group comparisons, Gabbiadini et al.’s study utilized an app to calculate daily steps, calories burned, and time spent active, and users were notified of their daily achievements directly on their smartphones [25]. The results demonstrated that the intervention group walked more (*P* = 0.02). Similarly, a study by Pope et al. reported an increase in MVPA at both week 5 and week 10 compared to baseline.

In addition, regarding PA-related psychosocial variables, only the study by Simons et al. compared between groups and found no significant changes in all indicators. For within-group comparisons, the intervention group showed a significant improvement in PA knowledge but a significant decrease in perceived benefits after the intervention. The study by Pope et al. found a 21% decrease in perceived impairment after the intervention in the intervention group. Gabbiadini et al. found significant improvements in attitudes and perceived behavioral control (PBC) (Table 4).

*3*.*3*.*1*.*2 Health app*. Three studies were included in this review to examine the impact of health apps on PA in healthy young adults (Table 3). Among the two studies that were compared between groups, no significant improvement was found in any of the PA-related variables. Kuston et al. developed a prototype of a technology-supported PA course based at a public university, which involved the development of a technical prototype based on seven initial design principles. During this phase, a prototype learning management system called Physical Education Supporting Site for Physical Activity (PESSPA) was designed, validated, and built, which was then integrated into a smartphone application called Physical Education Supporting App for Physical Activity (PESAPA) [26]. The results showed that the technology-supported physical education course positively influenced PA (i.e., MVPA, steps), although the improvements did not reach a significant level. In Epton et al.’s study, participants were asked to use an app and access online resources containing theory-based information related to each of the four target health behaviors (i.e., text, videos and links to further information) and a planner to facilitate intention formation for implementing four target health behaviors [24]. The results indicated no differences in PA (i.e., nighttime activity, walking activity, university sports center membership and use) between the intervention and control groups. However, within-group comparisons in Wang et al.’s study showed that the participants who used the WeChat app and its Zhishi mini-program for health education, diet, and PA monitoring exhibited an enhanced PA level [29]. Among the 48 participants with low PA levels at baseline, 26 of them moved to a higher level after the 21-day intervention (*P* = 0.004). In summary, the overall findings suggest that health apps had no significant effect on improving PA in healthy young adults.

In terms of psychological outcomes related to PA, the study by Kuston et al. found that small-to-medium effect sizes favoring the intervention group in motivation outcomes and PA knowledge. However, the other studies did not specifically mention this outcome variables (Table 4).

#### 3.3.2 Combining p values

**Fig 2:**
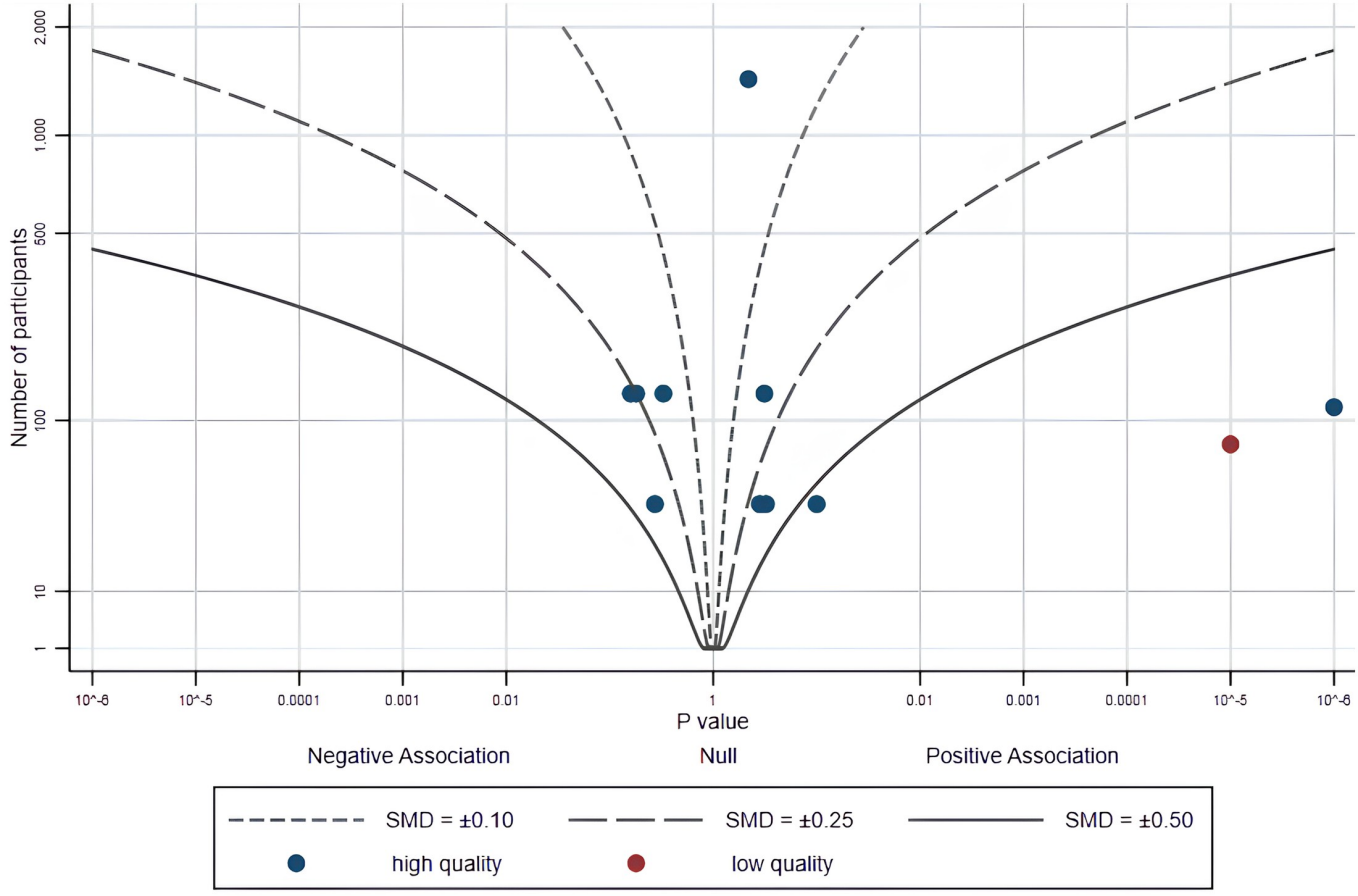


Six (11 effects) of the 8 studies provide a precise p value and direction of effect (S4 Table). We provide the albatross plot for these p values and corresponding study sample sizes in Fig 2. Most studies fall between contour lines of 0.1 and 0.5. The sample size of most studies is similar, with around 100 participants. However, these points have some noticeable dispersion on different contour lines. More than half of the research results showed a positive effect, but no significant difference was observed. Two studies have shown significant positive effects based on smartphone apps (*p* < 0.001), with one study being a low-risk, high-quality study. The results related to PA psychological correlates are less than 5 data points, making it impossible to use combining p values.

**Fig 3:**
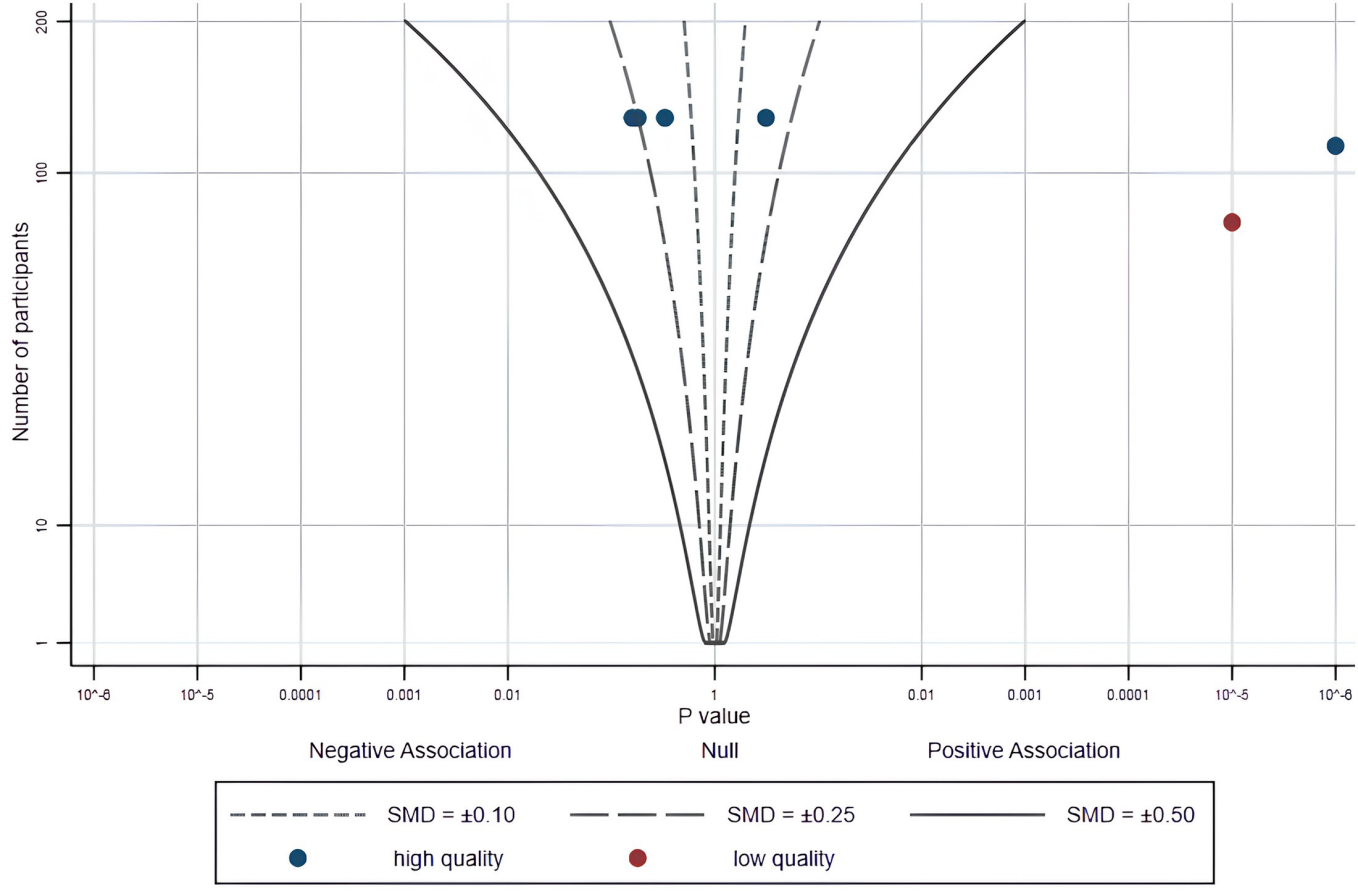


Four studies were included in this review to explore the effects of using fitness app on PA in healthy young adults. Three (6 effects) of the 4 studies provide a precise p value and direction of effect (S4 Table). We provide the albatross plot for these p values and corresponding study sample sizes in Fig 3. Most studies fall between contour lines of 0.1 and 0.25. The sample size of most studies is similar, with around 100 participants. Half of the studies showed positive effects, with two studies showing significant positive effects based on smartphone apps (*p* < 0.001), one of which was a low-risk, high-quality study.

**Fig 4:**
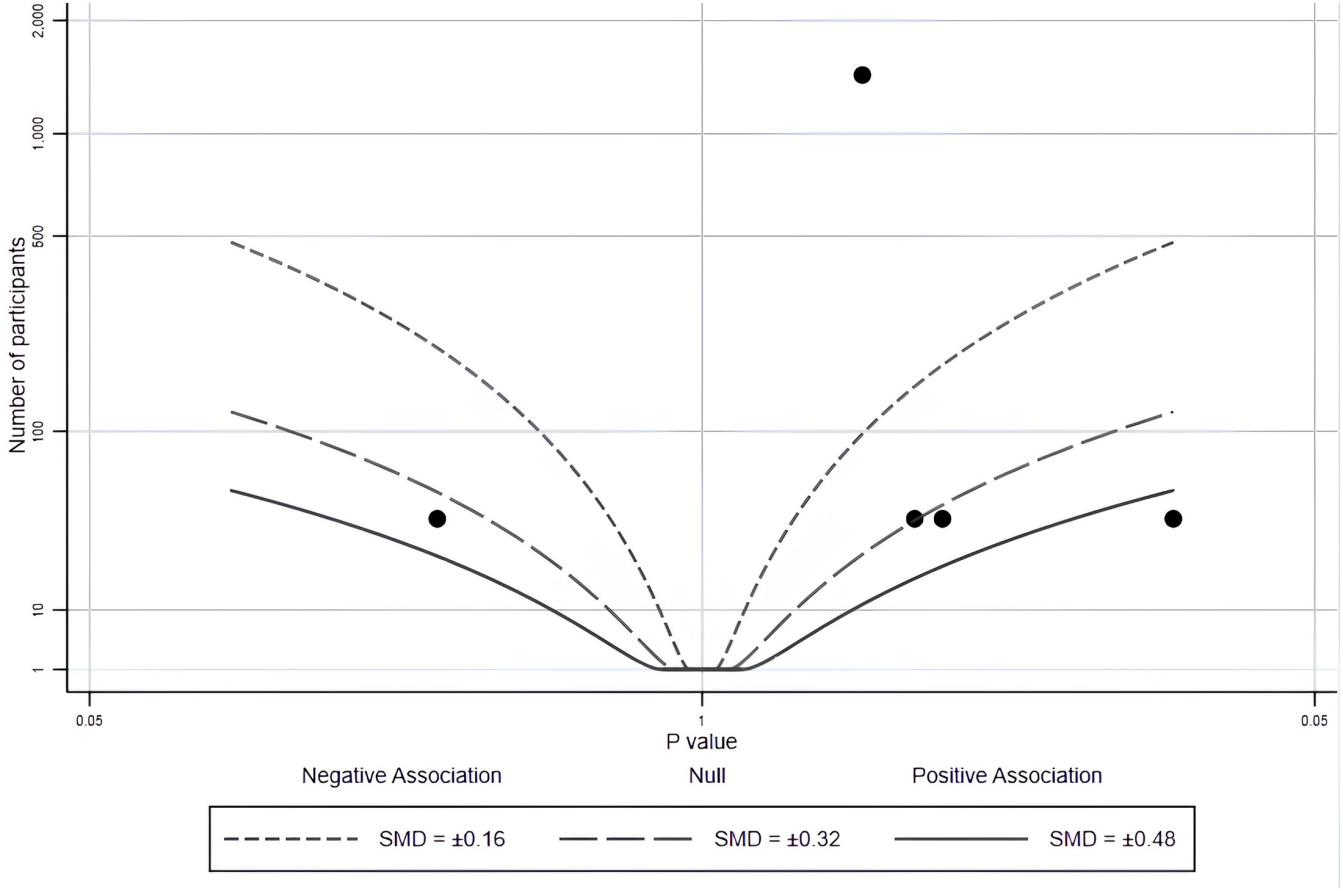


Three studies were included in this review to explore the effects of using health app on PA in healthy young adults. Two (5 effects) of the 3 studies provide a precise p value and direction of effect (S4 Table). We provide the albatross plot for these p values and corresponding study sample sizes in Fig 4. Most studies have shown positive effects, but none of the studies showed significant effects (*p* > 0.05).

#### 3.3.3 Assessing risk of reporting bias

Regarding the study limitations and the assessment of bias and quality (as seen in Table 2), the majority of studies were evaluated as having a low risk of bias, indicating no significant limitations that would necessitate downgrading. Specifically, issues of selective outcome reporting were noted, such as Gabbiadini’s study omitting baseline data, raising concerns over selective reporting. Similarly, Wang’s study, which utilized the International Physical Activity Questionnaire for PA assessment, only disclosed select significant findings. Considering the potential for publication bias, the studies generally had consistent subject sizes and intervention effects. However, this research was limited to English-language publications, excluding outcomes in other languages, which might introduce publication bias.

## 4. Discussion

The aim of this study was to synthesize the effects of smartphone-based app interventions on improving PA behaviors and psychological correlates in healthy young adults. For young adults, lack of PA has become a significant risk factor for health [43–45]. Using an intervention that is easy to implement and can be easily integrated into their busy lives may be more effective than finding extra time for recreational PA. Smartphone apps offer convenience and may serve as a promising intervention tool.

The findings of this study indicated that the app-based intervention did not yield a significant improvement in enhancing physical activity (PA) and PA-related psychosocial variables. Only a few of the included studies demonstrated improvements in the intervention group following the intervention. This suggests that the app may lack an effective mechanism for long-term motivation. A systematic review and meta-analysis conducted by Silva et al. supported the notion that short-term app-based interventions were effective in increasing the number of steps among adults [19]. Similarly, Romeo et al.’s study showed that app interventions lasting less than 3 months were more effective than longer interventions [21]. These findings could be attributed to the initial novelty of the app, which may have initially motivated the participants to adhere to the implementation plan. However, as the novelty wore off, their interest and dedication may have declined. Additionally, the app may not be adequately tailored to the specific needs of young users, who may require advanced contextual awareness in addition to simple goal feedback [46, 47].

However, the results of this study should be interpreted with caution due to the high heterogeneity among the included studies, which were also limited in number. Furthermore, nearly half (3 out of 7) of the included studies exhibited a low-quality study design. These findings align with the conclusions drawn by Marcolino et al. in their investigation of the effects of mHealth interventions on health [48], as well as the studies conducted by Silva et al. [19] and Rodríguez-González et al. [16], which explored the effects of app interventions on PA. These observations underscore the necessity for more high-quality research to address the prevalence of low-quality interventions and provide clarity on numerous aspects. Encouragingly, notable improvements were observed in terms of reducing barriers to PA and enhancing knowledge, and overall, the retention rates were high, with participants expressing satisfaction with their participation. This suggests that while current app-based interventions may not be sufficient to improve PA, the advantages of mobile phone apps, such as their user-friendly and self-explanatory features, as well as their simplicity, hold potential for providing intervention strategies to enhance PA behaviors and related psychosocial outcomes.

### 4.1. Fitness apps

Currently, there is an abundance of commercial fitness apps available, with estimates suggesting there are at least one million of them [49]. The accessibility and user-friendly nature of these apps make them a valuable tool for improving PA levels in the population. In this analysis, we focused on four studies that examined fitness app interventions to enhance PA in young, healthy adults. One study reported a significant increase in step count, another study found no significant change, and the remaining two studies did not include between-group comparisons. These findings align with the results of Romeo et al. [21], who conducted a systematic review and meta-analysis aiming to evaluate the effectiveness of smartphone apps in increasing PA among adults. Their findings indicated that smartphone apps did not lead to a significant increase in the average number of steps taken by participants.

In contrast, Kim et al. conducted a systematic review and meta-analysis to assess the impact of a mobile smartphone-based health program on PA and obesity outcomes specifically in young adults [17]. Their findings revealed that the smartphone app intervention had a significant effect on weight loss and increased PA. The disparities between the results of these studies may be attributed to variations in the study populations. For instance, Romeo et al. [21] examined a more diverse population of individuals aged 18 years and older, including those with various medical conditions. On the other hand, Kim et al. specifically targeted individuals aged 19 to 35 years with overweight and obesity, which might have led to more favorable outcomes for individuals with specific chronic conditions [17]. In the current study, our participants consisted of healthy individuals aged 18–35 years, and it is possible that the smartphone app intervention did not result in significant improvement within this healthy population when compared to those with underlying diseases. However, it is important to interpret these findings cautiously due to the limited number of included studies, which may limit the generalizability of the results.

### 4.2. Health apps

This review examined three studies that utilized a health app intervention to enhance PA in young, healthy adults. The results of both studies did not show a significant improvement in PA-related outcomes. In Wang et al.’s study, 48 participants initially had a low level of PA, and 26 of them showed progress to a higher level after the 21-day intervention. Therefore, while the current health app may not have a substantial effect on improving PA in healthy young adults, it does demonstrate some ameliorative impact. One possible explanation is that the health app intervention incorporates components such as dietary nutrition and health education. As a result, participants may tend to overlook the completion of the PA intervention, which can influence the overall effectiveness of the intervention. Additionally, it is possible that the design of the health app’s PA interventions did not adequately influence and engage the participants.

This finding aligns with the systematic review conducted by Romeo et al., which found that apps focusing solely on PA were more effective compared to those combining PA with diet interventions. Similar results have been observed in previous research on wearable devices, where designs specifically addressing sedentary behavior (SB) were more effective than those targeting both PA and SB [50]. By analogy, an app that specifically targets PA may have a greater impact compared to an app that encompasses multiple health intervention goals.

### 4.3. Strengths and limitations

This study possesses several strengths. Firstly, it is the first documented systematic review focusing on smartphone app-based interventions to enhance physical activity (PA) and PA-related psychological correlates in healthy young adults. Secondly, the study strictly adhered to the PRISMA guidelines in terms of the research process and report writing, providing a valuable reference for future investigations in this field. Thirdly, the study categorized the interventions into fitness apps and health apps based on their characteristics and conducted relevant analyses, offering significant insights for the advancement of theoretical understanding and practical application of mobile app-based interventions. However, there are several limitations to consider in this study. Firstly, the number of included studies was relatively small, and substantial heterogeneity was observed among them, making it challenging to perform a meta-analysis. Secondly, most of the studies had short intervention periods, and the long-term effects of these interventions remain unclear. Finally, the intervention strategies varied across the studies, preventing definitive conclusions about the most effective intervention approach. These limitations are in line with the findings of a recent umbrella review by Rodríguez-González et al. on app interventions for PA, which emphasized the importance of considering elements such as intervention components, context/environment/setting, intervention duration, and target population in future studies [16].

## 5. Conclusion

The findings of this study indicate that current mobile app-based interventions may not be effective in improving PA behavior and PA-related psychological correlates in healthy young adults (aged 18–35 years). However, there is a noticeable trend towards improvement, particularly in areas such as general health and PA knowledge. It is important to acknowledge that due to the limited number of studies included in this review, further research is required to determine the true effects of these interventions. Therefore, definitive conclusions cannot be drawn solely from this review. Future investigations should employ rigorous study designs to explore intervention effects in greater detail and examine different types of mobile app interventions, including variations in app characteristics (e.g., fitness app and health app), combinations of interventions (e.g., fitness app alone and fitness app combined with other components), diverse intervention goals (e.g., PA and PA combined with other outcomes), and various intervention characteristics such as frequency and periodicity. Conducting in-depth research in these areas is crucial for obtaining a comprehensive understanding of the potential benefits of mobile app interventions in promoting PA and related outcomes among young, healthy adults.

## Supporting information

S1 Table PRISMA checklist. (DOCX)

S2 Table Search strategy. (DOCX)

S3 Table Items and response options relating to risk of reporting biases in GRADE. (DOCX)

S4 Table Raw data were converted into statistical data required for vote counting and combining P values. (DOCX)
