# Supplementary material for: Smartphone app-based interventions on physical activity behaviors and psychological correlates in healthy young adults: A systematic review
Source: PLoS One. 2024 Apr 5;19(4):e0301088. doi: 10.1371/journal.pone.0301088 (PMC10997080; doi:10.1371/journal.pone.0301088)
Supplement: S2 Table — (DOCX) [file pone.0301088.s002.docx]

**S2 Table. Search Strategies.**

| Database | Search strategy | Scope& Retrieval steps |
| --- | --- | --- |
| PubMed | (“app”[tiab] OR “apps”[tiab] OR “smartphone app*”[tiab] OR “mobile app*” OR “mobile health”[tiab] OR “mobile phone”[tiab] OR “smartphone”[tiab] OR “smart phone”[tiab] OR “cellphone”[tiab] OR “cell phone”[tiab] OR “mHealth”[tiab])  AND  (“physical activity”[tiab] OR exercis*[tiab] OR step*[tiab] OR inactivit*[tiab] OR “sedentary behavior*”[tiab] OR “screen time”[tiab] OR “screentime”[tiab] OR “screenlife”[tiab] OR “sitting time”[tiab]) | Title, Abstract, Age (19-44 years), Article type (randomized controlled trial, clinical trial) |
| Medline | (“app” OR “apps” OR “smartphone app*” OR “mobile app*” OR “mobile health” OR “mobile phone” OR “smartphone” OR “smart phone” OR “cellphone” OR “cell phone” OR “mhealth”)  AND  (“physical activity” OR exercis* OR step* OR inactivit* OR “sedentary behavior*” OR “screen time” OR “screenlife” OR “screentime”OR “sitting time”) AND (“randomized controlled trial” OR “clinical trial” OR random* OR trial) | Abstract, Age (19-44 years); |
| SPORTDiscus | (“app” OR “apps” OR “smartphone app*” OR “mobile app*” OR “mobile health” OR “mobile phone” OR “smartphone” OR “smart phone” OR “cellphone” OR “cell phone” OR “mhealth”)  AND  (“physical activity” OR exercis* OR step* OR inactivit* OR “sedentary behavior*” OR “screen time” OR “screen life” OR “screentime”OR “sitting time”) AND (“randomized controlled trial” OR “clinical trial” OR random* OR trial) | Abstract, Age (19-44 years) |
| Web of Science | TS = (“app” OR “apps” OR “smartphone app*” OR “mobile app*” OR “mobile health” OR “mobile phone” OR “smartphone” OR “smart phone” OR “cellphone” OR “cell phone” OR “mhealth”)  AND  TS = (“physical activity” OR exercis* OR step* OR inactivit* OR “sedentary behavior*” OR “screen time” OR “screenlife” OR “screentime” OR “sitting time”) AND TS=(“randomized controlled trial” OR “clinical trial” OR random* OR trial) AND TS=(adult* OR “young adult*” OR universit* OR college OR undergraduate* OR graduate* OR women OR worker* OR employee*) | Web of science core collection, English, Article |
| Scopus | TITLE-ABS-KEY ( ( "app" OR "apps" OR "smartphone app*" OR "mobile app*" OR "mobile health" OR "mobile phone" OR "smartphone" OR "smart phone" OR "cellphone" OR "cell phone" OR "mhealth" ) AND ("physical activity" OR exercis* OR step* OR inactivit* OR "sedentary behavior*" OR "screen time" OR "screenlife" OR "sitting time") AND ("randomized controlled trial" OR "clinical trial" OR random* OR trial) AND (adult* OR "young adult*" OR universit* OR college OR undergraduate* OR graduate* OR women OR worker* OR employee* ) ) AND PUBYEAR > 2006  AND  ( EXCLUDE ( DOCTYPE , "re" ) OR EXCLUDE ( DOCTYPE , "cp" ) OR EXCLUDE ( DOCTYPE , "no" ) OR EXCLUDE ( DOCTYPE , "le" ) OR EXCLUDE ( DOCTYPE , "er" ) OR EXCLUDE ( DOCTYPE , "cr" ) OR EXCLUDE ( DOCTYPE , "bk" ) OR EXCLUDE ( DOCTYPE , "ch" ) OR EXCLUDE ( DOCTYPE , "ed" ) )  AND  ( LIMIT-TO ( LANGUAGE , "English" ) ) | English |
| Academic Search Premier | ("app" OR "apps" OR "smartphone app*" OR "mobile app*" OR "mobile health" OR "mobile phone" OR "smartphone" OR "smart phone" OR "cellphone" OR "mhealth")  AND  ("physical activity" OR exercis* OR step* OR inactivit* OR "sedentary behavior*" OR "screen time" OR "screenlife" OR "sitting time") AND ("randomized controlled trial" OR "clinical trial" OR random* OR trial) AND (adult* OR "young adult*" OR universit* OR college OR undergraduate* OR graduate* OR women OR worker* OR employee*) | English |
| Communication and Mass Media Complete | ("app" OR "apps" OR "smartphone app*" OR "mobile app*" OR "mobile health" OR "mobile phone" OR "smartphone" OR "smart phone" OR "cellphone" OR "mhealth")  AND  ("physical activity" OR exercis* OR step* OR inactivit* OR "sedentary behavior*" OR "screen time" OR "screenlife" OR "sitting time") AND ("randomized controlled trial" OR "clinical trial" OR random* OR trial) AND  (adult* OR "young adult*" OR universit* OR college OR undergraduate* OR graduate* OR women OR worker* OR employee*) | English |
| ArticleFirst | ("app" OR "apps" OR "smartphone app" OR "mobile app" OR "mobile health" OR "mobile phone" OR "smartphone" OR "smart phone" OR "cellphone" OR "mhealth")  AND  ("physical activity" OR exercis* OR step* OR inactivit* OR "sedentary behavior*" OR "screen time" OR "screenlife" OR "sitting time") | Key words |
| BioOne | ("app" OR "apps" OR "smartphone app*" OR "mobile app*" OR "mobile health" OR "mobile phone" OR "smartphone" OR "smart phone" OR "cellphone" OR "mhealth")  AND  ("physical activity" OR exercis* OR step* OR inactivit* OR "sedentary behavior*" OR "screen time" OR "screenlife" OR "sitting time") AND ("randomized controlled trial" OR "clinical trial" OR random* OR trial) AND (adult* OR "young adult*" OR universit* OR college OR undergraduate* OR graduate* OR women OR worker* OR employee*) | Unlimited |
| EBSCOHost | ("app" OR "apps" OR "smartphone app*" OR "mobile app*" OR "mobile health" OR "mobile phone" OR "smartphone" OR "smart phone" OR "cellphone" OR "mhealth")  AND  ("physical activity" OR exercis* OR step* OR inactivit* OR "sedentary behavior*" OR "screen time" OR "screenlife" OR "sitting time") AND ("randomized controlled trial" OR "clinical trial" OR random* OR trial) AND (adult* OR "young adult*" OR universit* OR college OR undergraduate* OR graduate* OR women OR worker* OR employee*) | Abstract |
| JSTOR | ((("app" OR "mobile health" OR "mobile phone" OR "smartphone" OR "cellphone" OR "mhealth")  AND  ("physical activity" OR step OR "sedentary behavior*" OR "screen time" OR "sitting time")) AND ("randomized controlled trial" OR "clinical trial")) | N/A |
| ProQuest | ("app" OR "apps" OR "smartphone app*" OR "mobile app*" OR "mobile health" OR "mobile phone" OR "smartphone" OR "smart phone" OR "cellphone" OR "mhealth")  AND  ("physical activity" OR exercis* OR step* OR inactivit* OR "sedentary behavior*" OR "screen time" OR "screenlife" OR "sitting time") AND ("randomized controlled trial" OR "clinical trial" OR random* OR trial) AND (adult* OR "young adult*" OR universit* OR college OR undergraduate* OR graduate* OR women OR worker* OR employee*) | N/A |
| SAGE Reference Online | ("app" OR "apps") AND ("physical activity" OR step* OR "sedentary behavior*")  AND  ("randomized controlled trial" OR "clinical trial" OR random* OR trial) AND (adult* OR "young adult*" OR universit* OR college OR undergraduate* OR graduate* OR women OR worker* OR employee*) | Full text |
| SpringerLink | App*[ Full text] AND “physical activity” [Full text]  AND  (adult* OR universit* OR college OR undergraduate* graduate* OR women OR worker* OR employee*)[Full text] AND trail[ti] | Full text, title |
| ScienceDirect | (app OR "mobile phone" OR smartphone OR mhealth) AND  ("physical activity" OR step OR "sedentary behavior" OR "screen time") AND trial | Title, abstract, keywords |
| Biomed Central | (title#“app*” OR title#"mobile health" OR title#"mobile phone" OR title#"smartphone" OR title#"cellphone" OR title#"mhealth")  AND  (title#“physical activity” OR title#exercis* OR title#step* OR title#inactivit* OR title# “sedentary behavior*” OR title#“screen time” OR title#“screenlife” OR title#“screentime” OR title#“sitting time”) | Title |
| Taylor & Francis | ("app" OR "apps" OR "smartphone app*" OR "mobile app*" OR "mobile health" OR "mobile phone" OR "smartphone" OR "smart phone" OR "cellphone" OR "mhealth")  AND  ("physical activity" OR exercis* OR step* OR inactivit* OR "sedentary behavior*" OR "screen time" OR "screenlife" OR "sitting time") AND ("randomized controlled trial" OR "clinical trial" OR random* OR trial) AND ( adult* OR "young adult*" OR universit* OR college OR undergraduate* OR graduate* OR women OR worker* OR employee*) | Abstract |
| Wiley Online | "app" OR "apps" OR "smartphone app*" OR "mobile app*" OR "mobile health" OR "mobile phone" OR "smartphone" OR "smart phone" OR "cellphone" OR "mhealth")  AND  ("physical activity" OR exercis* OR step* OR inactivit* OR "sedentary behavior*" OR "screen time" OR "screenlife" OR "sitting time" | Title |
