# Supplementary material for: Smartphone app-based interventions on physical activity behaviors and psychological correlates in healthy young adults: A systematic review
Source: PLoS One. 2024 Apr 5;19(4):e0301088. doi: 10.1371/journal.pone.0301088 (PMC10997080; doi:10.1371/journal.pone.0301088)
Supplement: S3 Table — (DOCX) [file pone.0301088.s003.docx]

S3 Table Items and response options relating to risk of reporting biases in GRADE

| Tool | Items | Response options |
| --- | --- | --- |
| GRADE[1-5] | 1. Study limitations (including selective outcome reporting) | **Study limitations domain – No serious limitations, do not downgrade:** Most information is from studies at low risk of bias (i.e. those with low risk of bias for all key criteria, including lack of allocation concealment, lack of blinding, incomplete accounting of patients and outcome events, selective outcome reporting bias, other limitations [stopping early for benefit, use of unvalidated outcome measures, carryover effects in crossover trial, recruitment bias in cluster-randomized trial])  **Study limitations domain – Serious limitations, rate down one level (i.e., from high to moderate quality):** Most information is from studies at moderate risk of bias  **Study limitations domain – Very serious limitations, rate down two levels (i.e., from high to low quality or moderate to very low):** Most information is from studies at high risk of bias. Selective reporting is present if authors acknowledge prespecified outcomes that they fail to report or report outcomes incompletely such that they cannot be included in a metaanalysis. One should suspect reporting bias if the study report fails to include results for a key outcome that one would expect to see in such a study or if composite outcomes are presented without the individual component outcomes. |
|  | 2. Publication bias | **Publication bias domain – Undetected:** None of the criteria for “strongly suspected” are met.  **Publication bias domain – Strongly suspected:** “In general, review authors and guideline developers should consider rating down for likelihood of publication bias when the evidence consists of a number of small studies. The inclination to rate down for publication bias should increase if most of those small studies are industry sponsored or likely to be industry sponsored (or if the investigators share another conflict of interest). Another criterion for publication bias is the pattern of study results. Suspicion may increase if visual inspection demonstrates an asymmetrical rather than a symmetrical funnel plot or if statistical tests of asymmetry are positive. Although funnel plots may be helpful, review authors and guideline developers should bear in mind that visual assessment of funnel plots is distressingly prone to error. Enhancements of funnel plots may (or may not) help to improve reproducibility and validity associated with their use...Furthermore, systematic review and guideline authors should bear in mind that even if they find convincing evidence of asymmetry, publication bias is not the only explanation. For instance, if smaller studies suffer from greater study limitations, they may yield biased overestimates of effects. Another explanation would be that, because of a more restrictive (and thus responsive) population, or a more careful administration of the intervention, the effect may actually be larger in the small studies. More compelling than any of these theoretical exercises is authors’ success in obtaining the results of some unpublished studies and demonstrating that the published and unpublished data show different results. In these circumstances, the possibility of publication bias looms large. The risk of publication bias is probably larger for observational studies than for RCTs, particularly small observational studies and studies conducted on data collected automatically (e.g. in the electronic medical record or in a diabetes registry) or data collected for a previous study. In these instances, it is difficult for the reviewer to know if the observational studies that appear in the literature represent all or a fraction of the studies conducted, and whether the analyses in them represent all or a fraction of those conducted. In these instances, reviewers may consider the risk of publication bias as substantial” 6. “Guideline panels and authors of systematic reviews should consider the extent to which they are uncertain about the magnitude of the effect due to selective publication of studies and they may downgrade the quality of evidence by one level. Consider: study design (experimental vs. observational); study size (small studies vs. large studies); lag bias (early publication of positive results); search strategy (was it comprehensive?); asymmetry in funnel plot” 8. “Relevant content: whether publication bias is undetected or suspected; interpretation of funnel plot; comprehensiveness of the search strategies and methods to identify all available evidence; presence of small (often positive) studies with for profit interest. Indicate the reason publication bias is detected (e.g. asymmetrical funnel plot, small studies with positive results, suspected selective availability of data from published, or unpublished studies)” |

Reference

1. Guyatt GH, Oxman AD, Vist GE, et al. GRADE: an emerging consensus on rating quality of evidence and strength of recommendations. BMJ 2008;336(7650):924-6.

2. Guyatt GH, Oxman AD, Montori V, et al. GRADE guidelines: 5. Rating the quality of evidence—

publication bias. J Clin Epidemiol 2011;64(12):1277-82.19.

3. Guyatt GH, Oxman AD, Vist G, et al. GRADE guidelines: 4. Rating the quality of evidence--study limitations (risk of bias). J Clin Epidemiol 2011;64(4):407-15.

4. Schünemann H, Brożek J, Guyatt G, et al. Handbook for grading the quality of evidence and the

strength of recommendations using the GRADE approach. [Updated October 2013]. Available from http://gdt.guidelinedevelopment.org/app/handbook/handbook.html.

5. Santesso N, Carrasco-Labra A, Langendam M, et al. Improving GRADE evidence tables part 3: detailed guidance for explanatory footnotes supports creating and understanding GRADE certainty in the evidence judgments. J Clin Epidemiol 2016.
