# Supplementary material for: Smartphone app-based interventions on physical activity behaviors and psychological correlates in healthy young adults: A systematic review
Source: PLoS One. 2024 Apr 5;19(4):e0301088. doi: 10.1371/journal.pone.0301088 (PMC10997080; doi:10.1371/journal.pone.0301088)
Supplement: S4 Table — (DOCX) [file pone.0301088.s004.docx]

S4 Table Raw data were converted into statistical data required for vote counting and combining P values

|  |  |  |  |  |  |  | Summary statistics | Combining P values | | Vote counting |
| --- | --- | --- | --- | --- | --- | --- | --- | --- | --- | --- |
| Study | Sample(N) | Type of app | Duration | Age | Outcome | Quality(scores) | Available data*** | Available data (2-sided P value) | Stand. metric (1-sided P value) | Available data*** |
| kuston et al., (2023) | I (n=22); C (n=17) | health app | 6 weeks | university students | TPA | 3 | I 1460.1(4859.2); C 702.3 (2525.3) | MD 757.8; P = 0.617 | 0.309 | MD 757.8; NS |
|  |  |  |  |  | VPA | 3 | I 1078.5(3315.3); C 334.3 (936.5) | MD 744.2; P =0.708 | 0.354 | MD 744.2; NS |
|  |  |  |  |  | MPA | 3 | I 420.4(1648.3); C -39.3 (1390.5) | MD 459.7; P = 0.214 | 0.1 | MD 459.7; NS |
|  |  |  |  |  | walking activity | 3 | I -38.9(1060.1); C 407.2 (944.6.5) | MD -368.3; P =0.548 | 0.274 | MD -368.3; NS |
|  |  |  |  |  | Amotivation | 3 | I -0.19(0.36); C 0.31 (0.89) | MD -0.5; P =0.02 | 0.01 | MD -0.5; Favours control |
|  |  |  |  |  | knowledge | 3 | I 3.64(4.42); C 1.91 (4.62) | MD 1.73; P =0.207 | 0.104 | MD 1.73; NS |
| Al-Nawaiseh et al. (2022) | I (n=56); C (n=58) | fitness app | 12 weeks | 21.12±2.2 | Daily step-count | 6 | I 14575.9(15809.1); C -730.48 (15989.8) | MD 15306.4; P < 0.001 | P < 0.001 | MD 15306.4; Favours intervention |
| Gabbiadini et al. (2019) | Total n=78 (69 female) | fitness app | 2 weeks | 19.94±1.36 | attitudes | 4 | I 6.45(0.37); C 6.17 (0.64) | Favours intervention, P < 0.001 | < 0.001 | MD = 0.28; Favours intervention |
|  |  |  |  |  | PBC scores | 4 | I 5.25(1.07); C 4.48(0.71) | Favours intervention, P < 0.001 | < 0.001 | MD = 0.77; Favours intervention |
|  |  |  |  |  | Walking activity | 4 | I 4.18(1.02); C 3.38(1.07) | Favours intervention, P = 0.001 | < 0.001 | MD = 0.8; Favours intervention |
| Wang et al. (2020) | I n=87 (9 female); C n=23 (10 female) | health app | 21 days | 22 ± 2 and 21 ± 4 | Number of low PA | 4 | — | low PA population -26 (48), P = 0.004 | 0.002 | Favours intervention |
| Pope et al. (2020) | I n=22 (16 female); C n=22(16 female) | fitness app | 10 weeks | 21.6 | MVPA | 5 | I -1.9(11.0); C -12.6(5.8) | MD -10.7 | — | NS |
|  |  |  |  |  | Self-Efficacy | 5 | Intervention -0.7(19.8); C 2.5(23.2) | MD -3.2 | — | NS |
|  |  |  |  |  | Social Support | 5 | I 21.0(43.8); C 15.3(39.8) | MD 5.7 | — | NS |
|  |  |  |  |  | Enjoyment | 5 | I 0.0(0.0); C 0.0(0.0) | MD 0 | — | NS |
|  |  |  |  |  | perceived Barriers | 5 | I 4.8(15.6); C 4.8(10.7) | MD 0 | — | NS |
|  |  |  |  |  | Outcome Expectancy | 5 | I 2.7(9.4); C 3.2(9.4) | MD -0.5 | — | NS |
| Simons et al. (2018) | I n=60 (25 female); C n=70 (42 female) | fitness app | 9 weeks | 25±3 | TPA | 8 | I -34(18.2); C -12.5 (18.4) | MD -21.5; P = 0.36 | 0.18 | MD -21.5; NS |
|  |  |  |  |  | MVPA | 8 | I -4.8(3.6); C -3.4 (3.9) | MD -1.4; P = 0.66 | 0.33 | MD -1.4; NS |
|  |  |  |  |  | LPA | 8 | I -29.1(16.7); C -9.2 (16.6) | MD -19.9; P = 0.31 | 0.16 | MD -19.9; NS |
|  |  |  |  |  | Daily step-count | 8 | I -878(614); C -921 (644) | MD 43; P = 0.64 | 0.32 | MD 43; NS |
|  |  |  |  |  | Benefits | 8 | I -0.3(0.1); C -0.2 (0.1) | MD -0.1; P = 0.75 | 0.38 | MD -0.1; NS |
|  |  |  |  |  | Barriers | 8 | I 0(0.1); C 0(0.1) | MD 0; P =0.82 | 0.41 | MD 0; NS |
|  |  |  |  |  | Self-efficacy | 8 | I 0(0.1); C 0(0.1) | MD 0; P =0.41 | 0.21 | MD 0; NS |
|  |  |  |  |  | Intention | 8 | I -0.2(0.2); C -0.1(0.2) | MD -0.1; P = 0.56 | 0.28 | MD -0.1; NS |
|  |  |  |  |  | Knowledge%(correct answer) | 8 | I 6.7%; C 14.2% | MD -7.5%; P = 0.51 | 0.26 | MD -7.5%; NS |
|  |  |  |  |  | Social Support | 8 | I -0.1(0.2); C 0.1(0.2) | MD -0.2; P = 0.25 | 0.13 | MD -0.2; NS |
| Epton et al., 2014 | I n=736 (453 female); C n=709 (391 female) | health app | 24 weeks | 18.9 | TPA | 8 | I 210.4(4637.8); C -86.3 (5122.9) | MD 296.7, P = 0.914 | 0.457 | MD 296.7; NS |

Note:

* All scales operate in the same direction. Higher scores indicate greater satisfaction.

** For a particular scenario, the ‘available data’ column indicates the data that were directly reported, or were calculated from the reported statistics, in terms of: effect estimate, direction of effect, confidence interval, precise P value, or statement regarding statistical significance (either statistically significant, or not).

direction = direction of effect reported or can be calculated; C = control group; MD = mean difference; NS = not statistically significant; I = intervention group.
